# Supplementary material for: Cleaning the Medicago Microarray Database to Improve Gene Function Analysis
Source: Plants (Basel). 2021 Jun 18;10(6):1240. doi: 10.3390/plants10061240 (PMC8234645; doi:10.3390/plants10061240)
Supplement: Supplementary file 1 [file plants-10-01240-s001.zip › FigureS1Marzorati-low-sum-exp.pptx]

## Slide 1
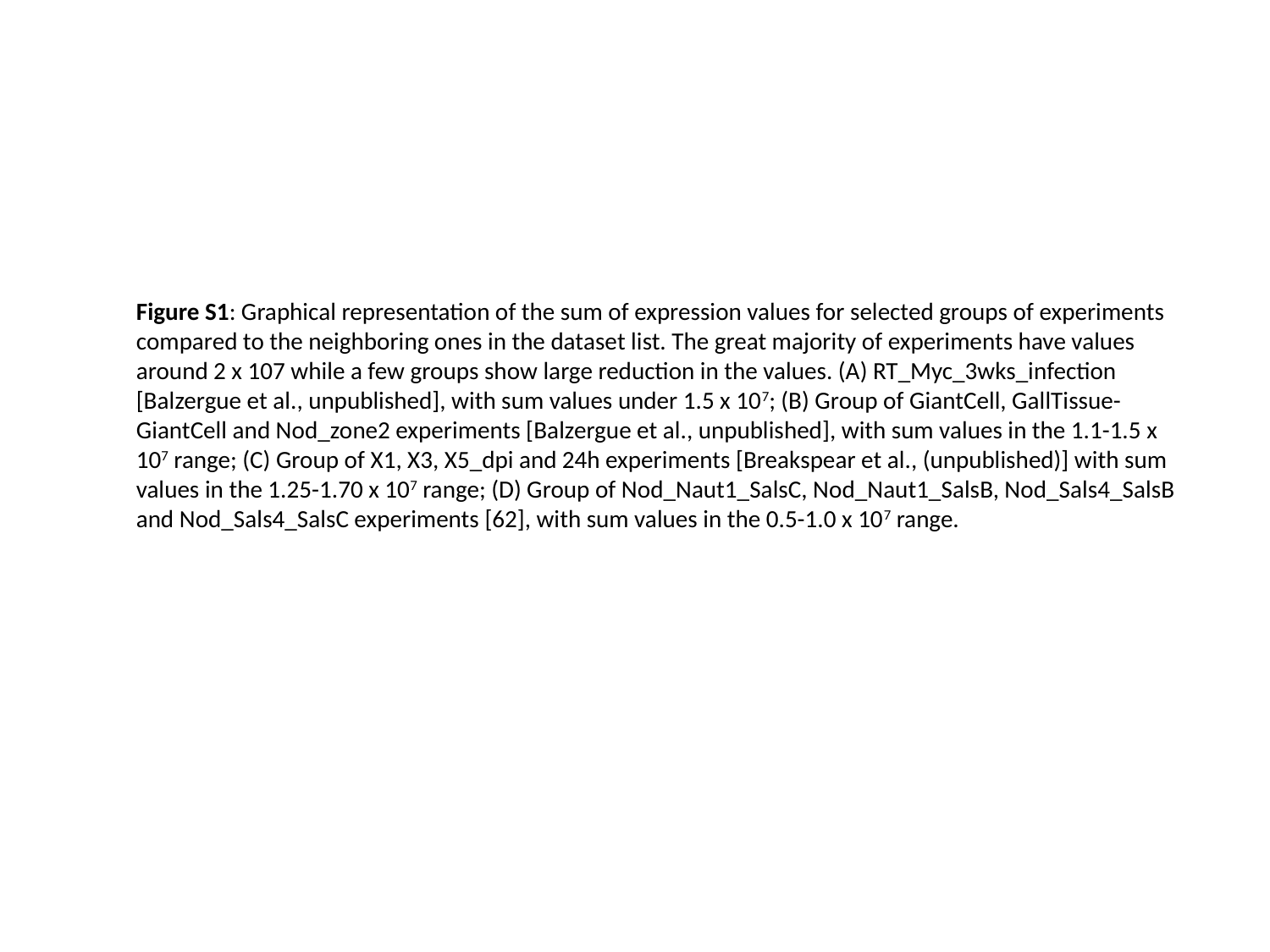

Figure S1: Graphical representation of the sum of expression values for selected groups of experiments compared to the neighboring ones in the dataset list. The great majority of experiments have values around 2 x 10^7^ while a few groups show large reduction in the values. (A) RT_Myc_3wks_infection [Balzergue et al., unpublished], with sum values under 1.5 x 10^7^; (B) Group of GiantCell, GallTissue-GiantCell and Nod_zone2 experiments [Balzergue et al., unpublished], with sum values in the 1.1-1.5 x 10^7^ range; (C) Group of X1, X3, X5_dpi and 24h experiments [Breakspear et al., (unpublished)] with sum values in the 1.25-1.70 x 10^7^ range; (D) Group of Nod_Naut1_SalsC, Nod_Naut1_SalsB, Nod_Sals4_SalsB and Nod_Sals4_SalsC experiments [62], with sum values in the 0.5-1.0 x 10^7^ range.

## Slide 2
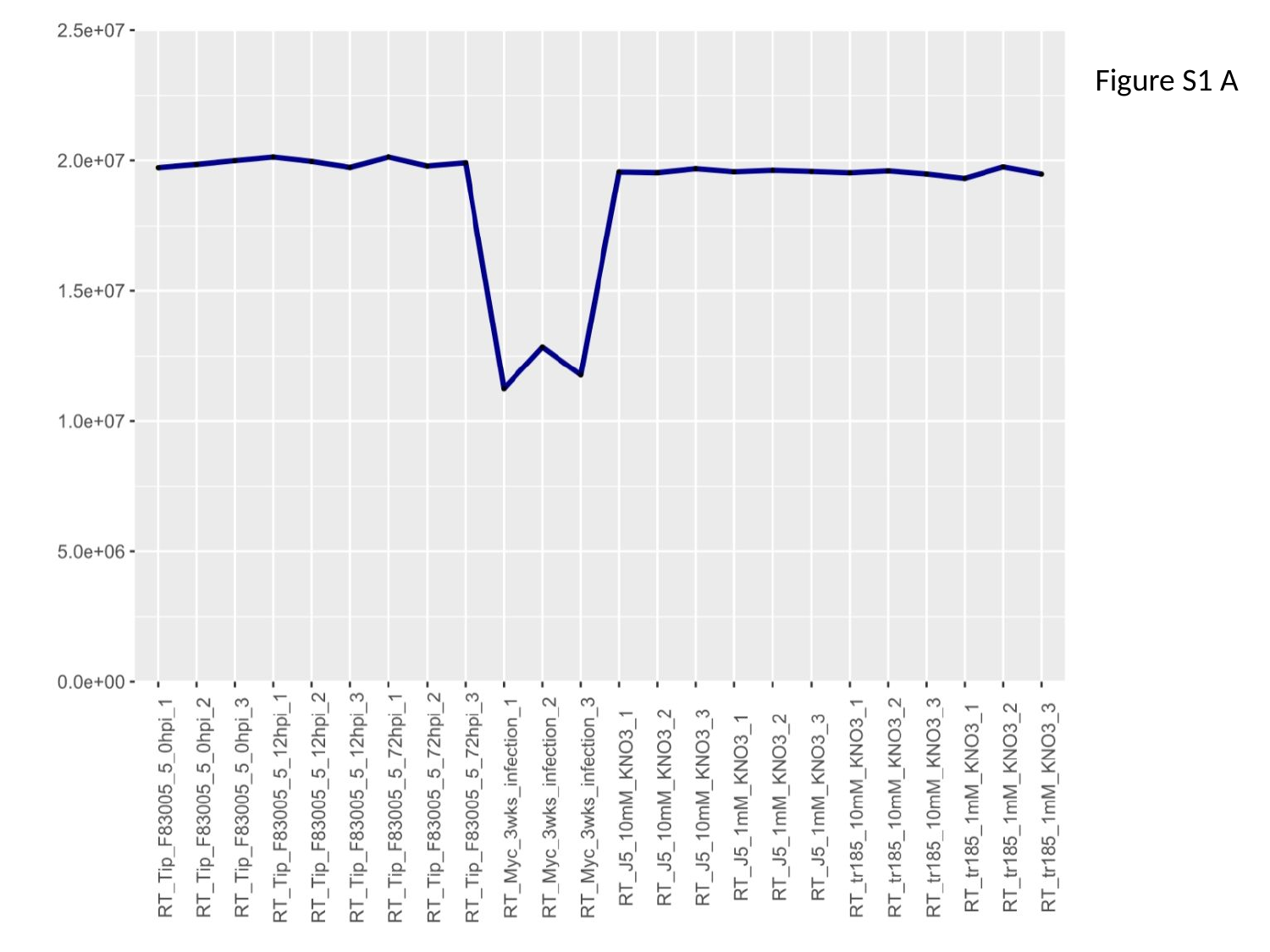

Figure S1 A

## Slide 3
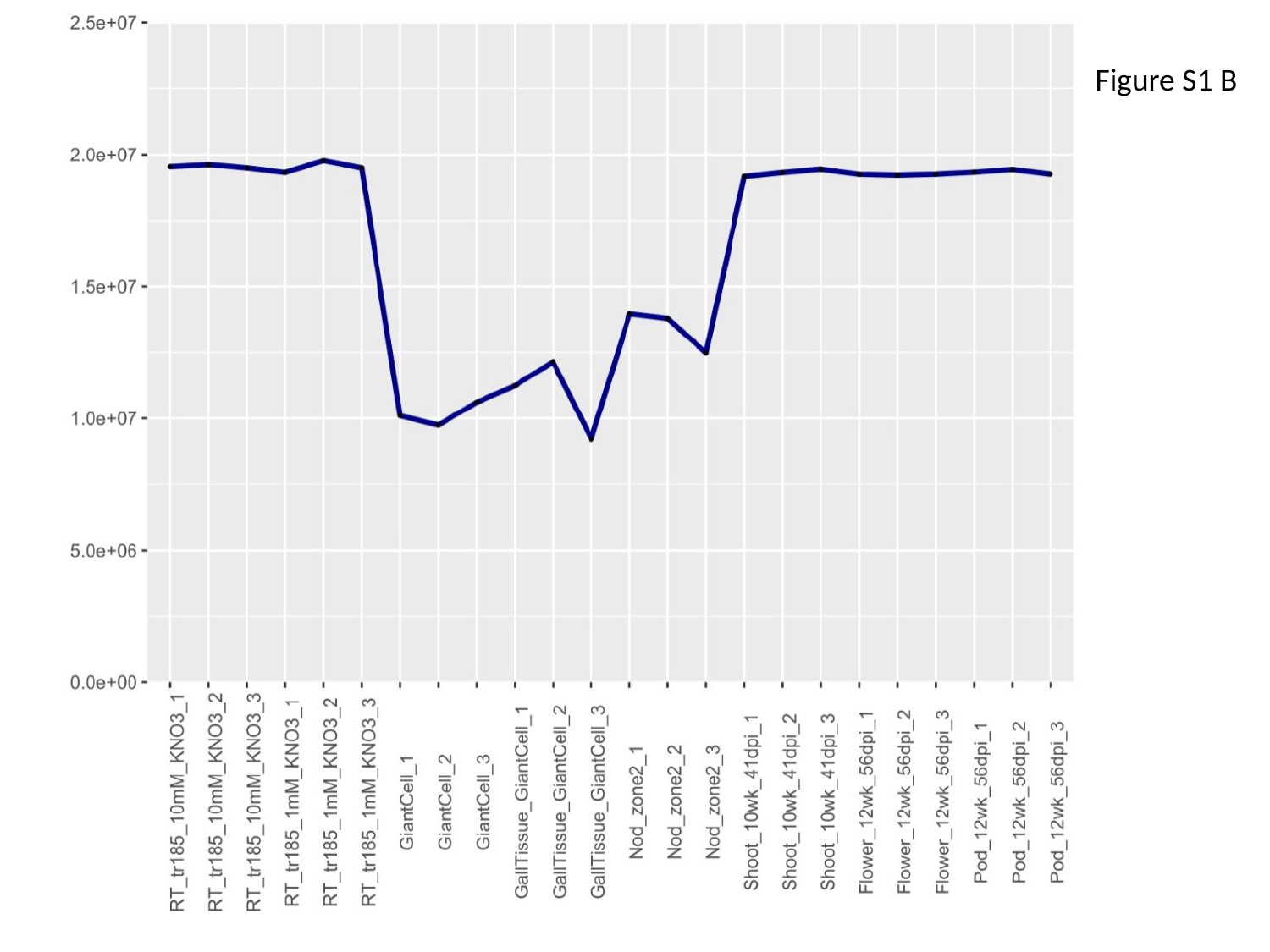

Figure S1 B

## Slide 4
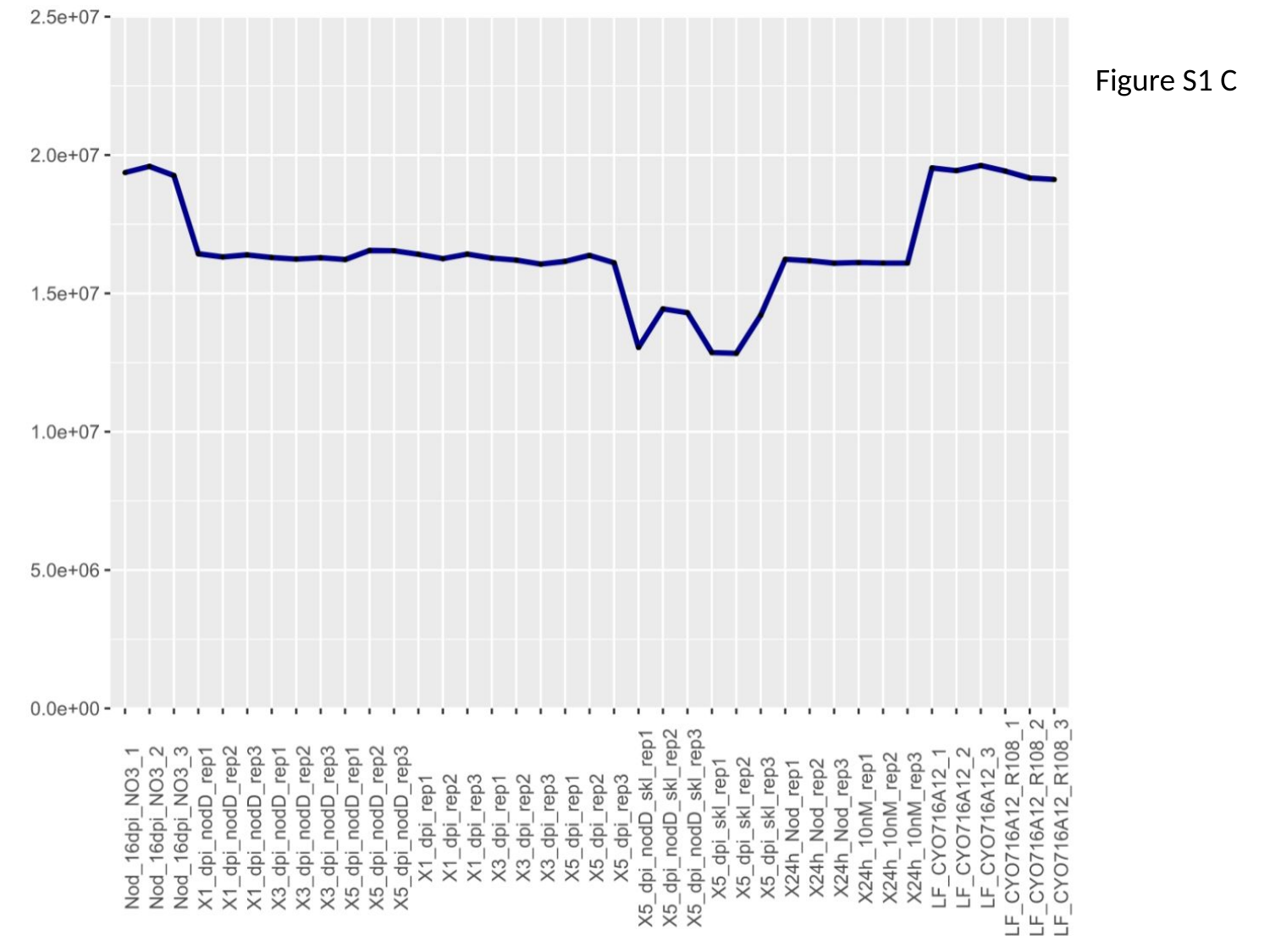

Figure S1 C

## Slide 5
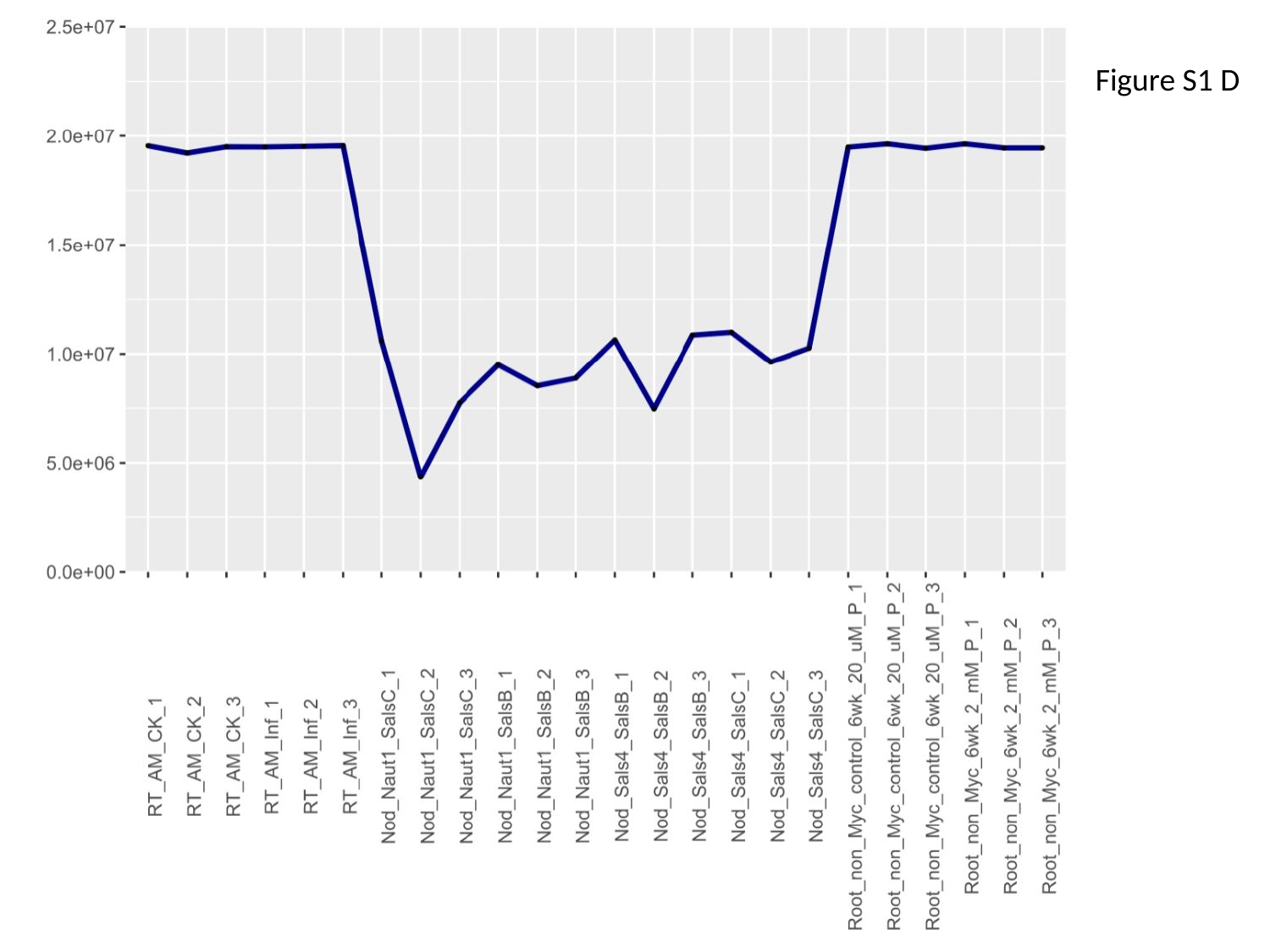

Figure S1 D
