# Supplementary material for: Cleaning the Medicago Microarray Database to Improve Gene Function Analysis
Source: Plants (Basel). 2021 Jun 18;10(6):1240. doi: 10.3390/plants10061240 (PMC8234645; doi:10.3390/plants10061240)
Supplement: Supplementary file 1 [file plants-10-01240-s001.zip › FigureS2-Marzorati_Scatterplots.pptx]

## Slide 1
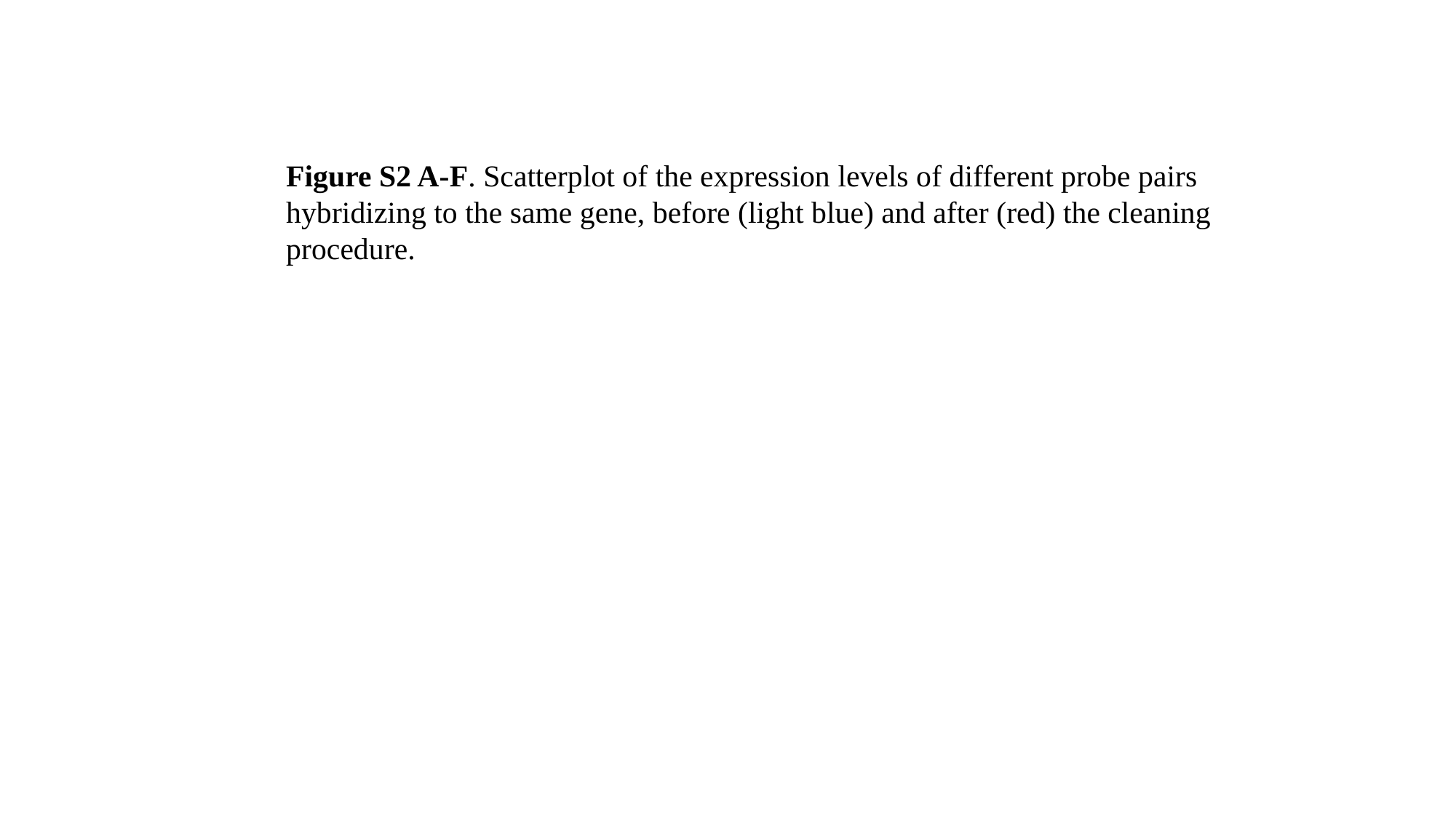

Figure S2 A-F. Scatterplot of the expression levels of different probe pairs hybridizing to the same gene, before (light blue) and after (red) the cleaning procedure.

## Slide 2
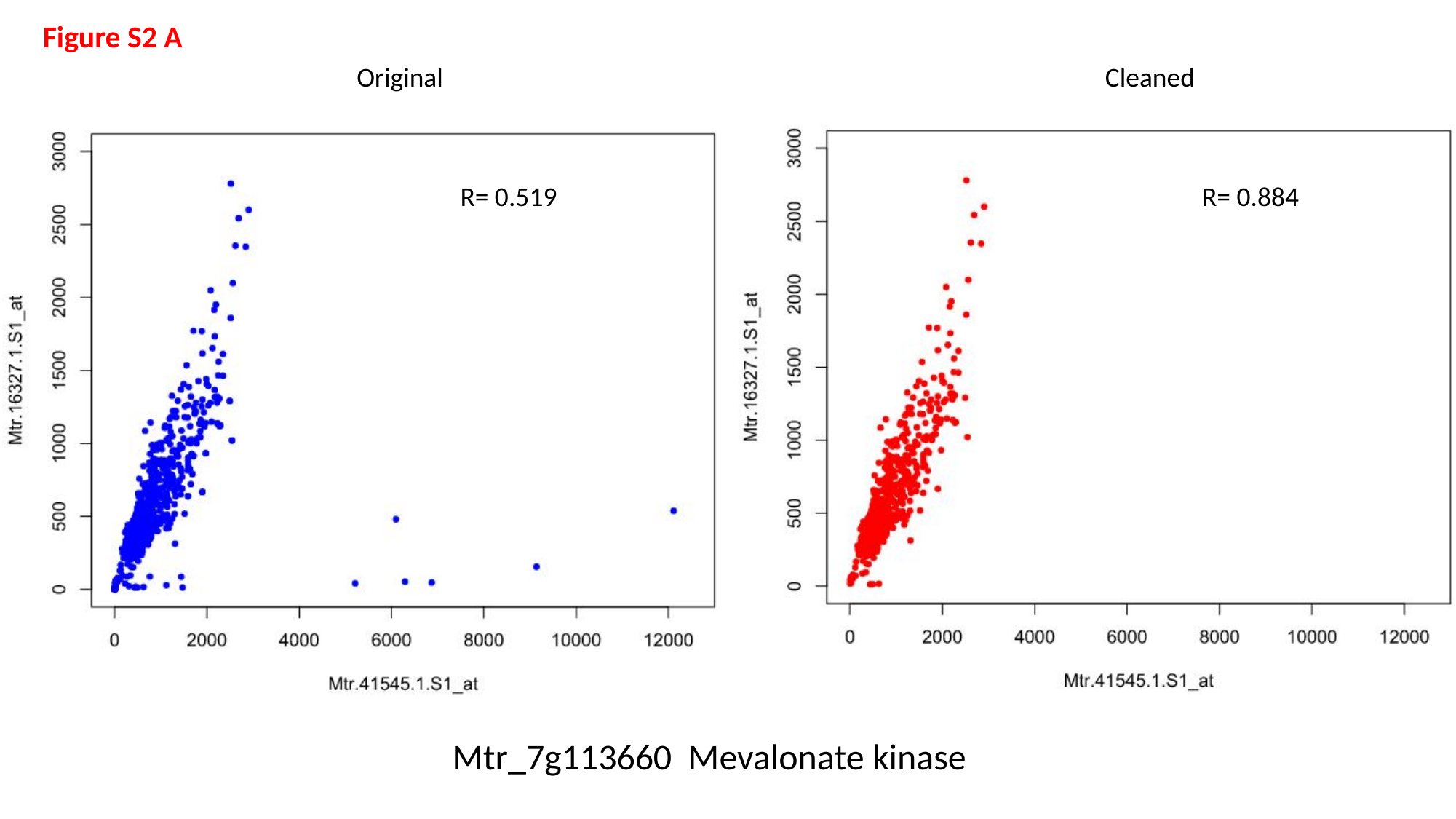

Figure S2 A
Original
Cleaned
R= 0.519
R= 0.884
Mtr_7g113660 Mevalonate kinase

## Slide 3
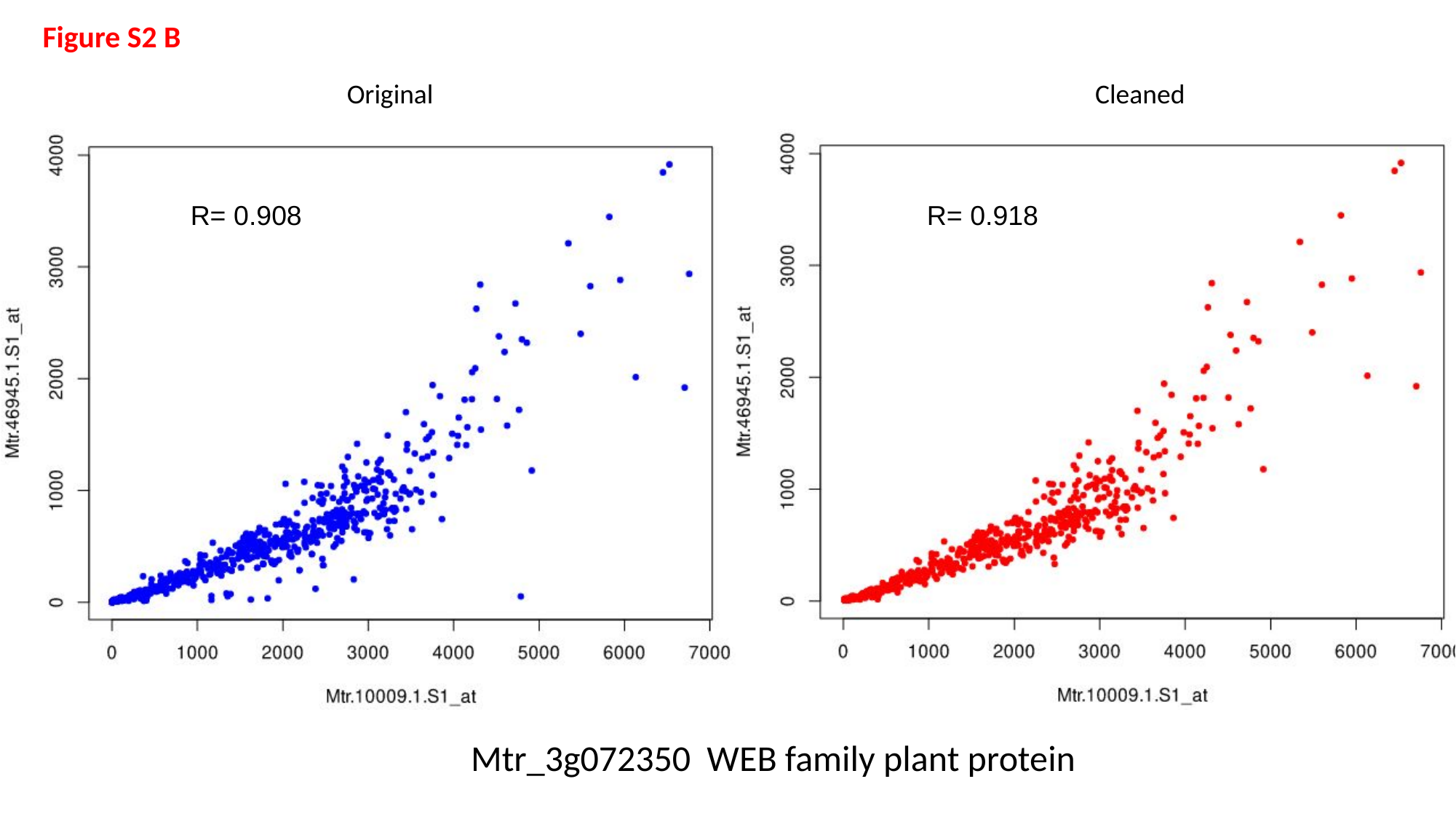

Figure S2 B
Original
Cleaned
R= 0.918
R= 0.908
Mtr_3g072350 WEB family plant protein

## Slide 4
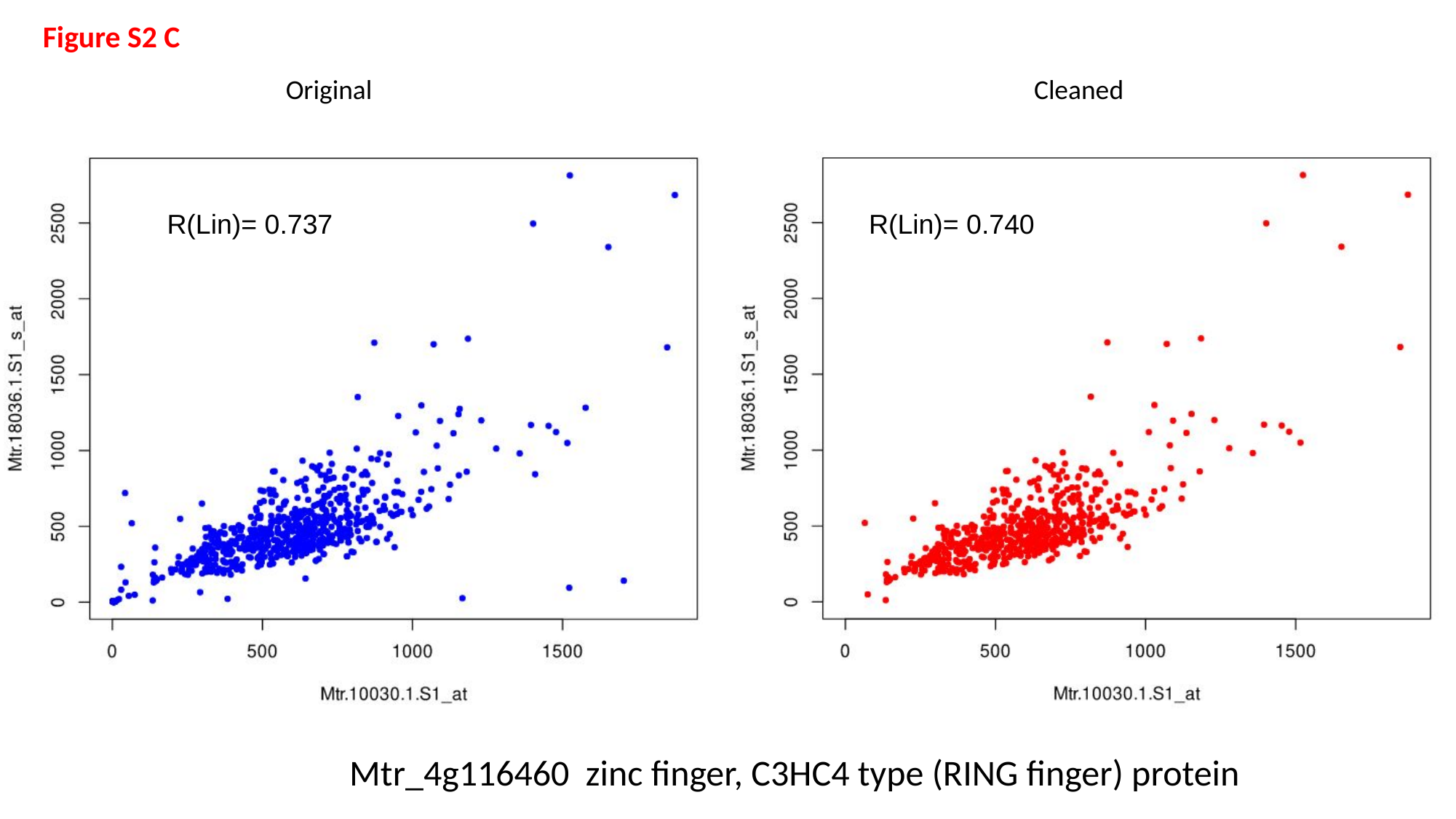

Figure S2 C
Original
Cleaned
R(Lin)= 0.737
R(Lin)= 0.740
Mtr_4g116460 zinc finger, C3HC4 type (RING finger) protein

## Slide 5
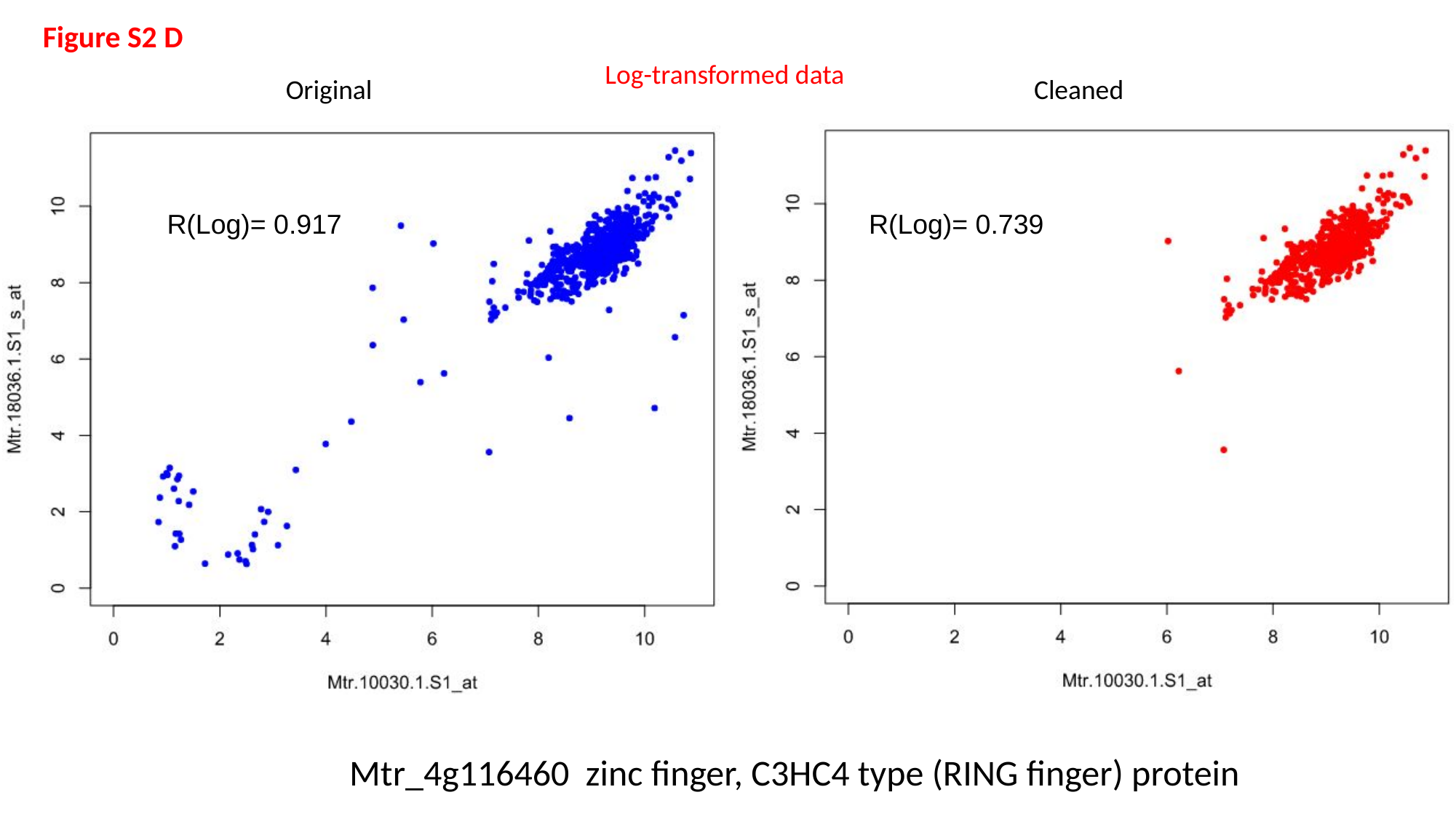

Figure S2 D
Log-transformed data
Original
Cleaned
R(Log)= 0.917
R(Log)= 0.739
Mtr_4g116460 zinc finger, C3HC4 type (RING finger) protein

## Slide 6
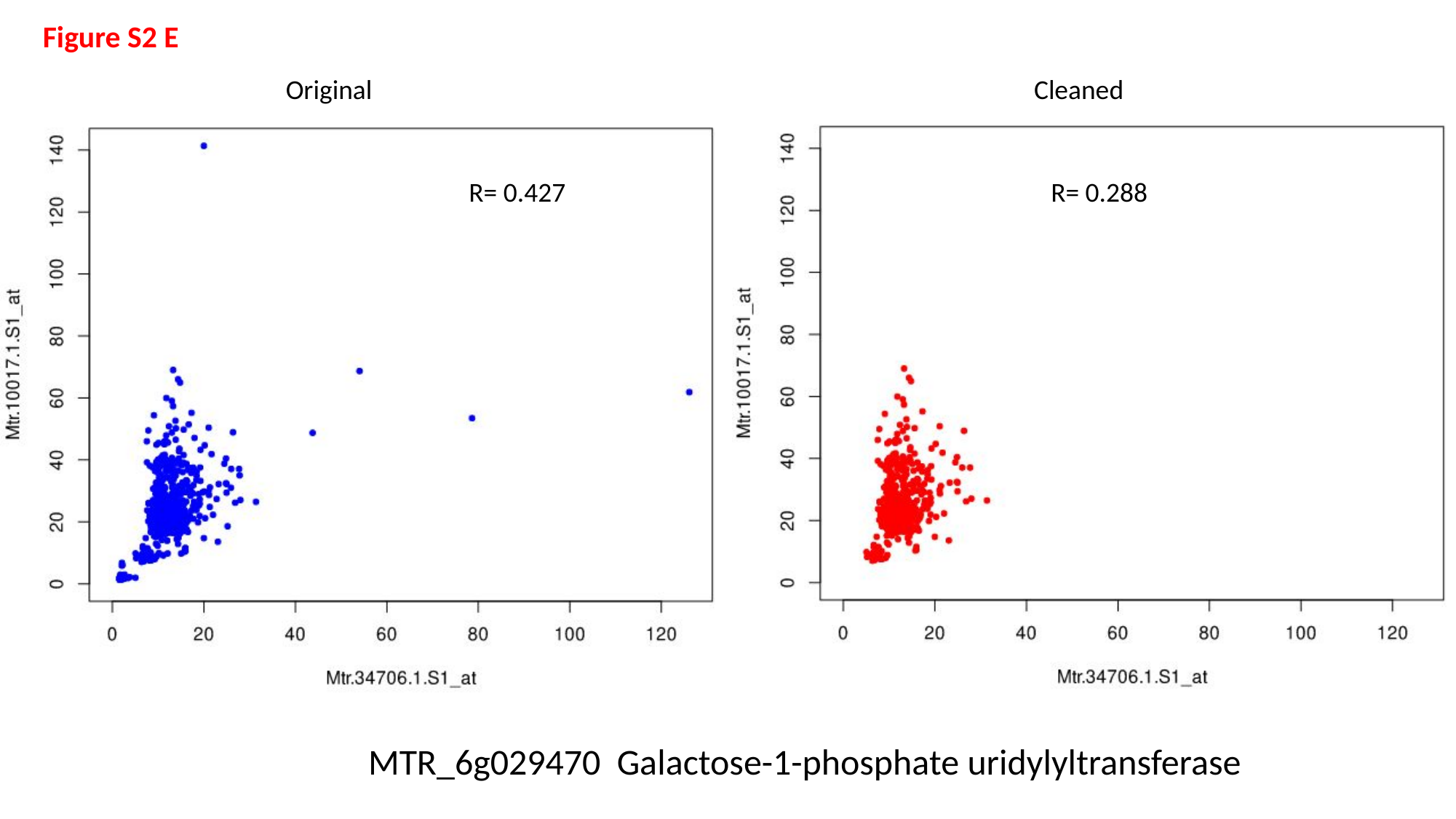

Figure S2 E
Original
Cleaned
R= 0.427
R= 0.288
MTR_6g029470 Galactose-1-phosphate uridylyltransferase

## Slide 7
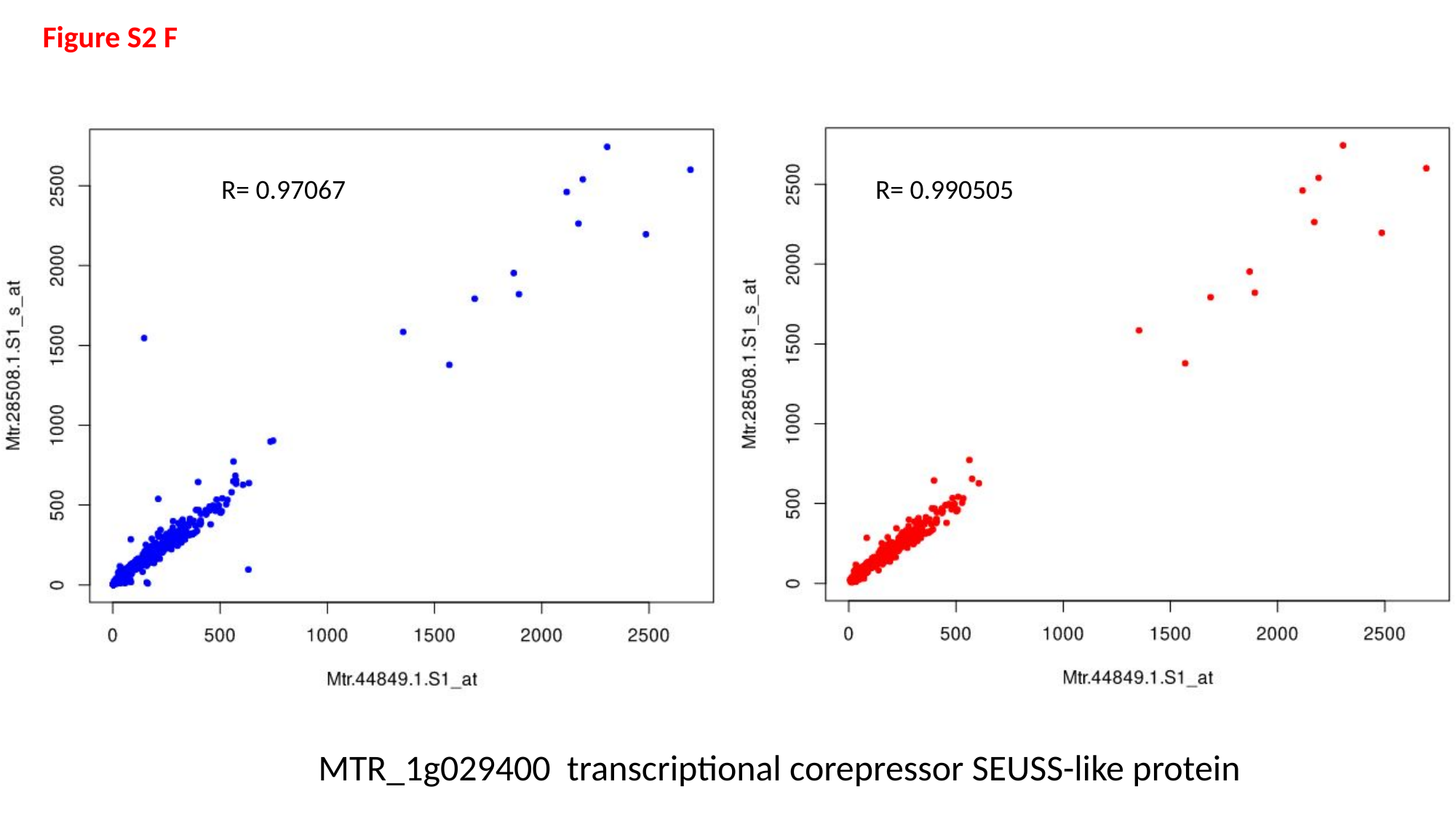

Figure S2 F
R= 0.97067
R= 0.990505
MTR_1g029400 transcriptional corepressor SEUSS-like protein
