# Supplementary material for: Cleaning the Medicago Microarray Database to Improve Gene Function Analysis
Source: Plants (Basel). 2021 Jun 18;10(6):1240. doi: 10.3390/plants10061240 (PMC8234645; doi:10.3390/plants10061240)
Supplement: Supplementary file 1 [file plants-10-01240-s001.zip › Table S04_Marzorati-Samples-Low-Sum-Values.pdf]

Table S4: Sum of the expression values for experiments of [31], [32] and [33]

A) Sum of the expression values for RT_LCM experiments [31] compared to the ones of RT_NFP_nsMyc_LCOs_6h, RT_NFP_sMyc_LCOs_6h, RT_NFP_s_nsMyc_LCOs_6h, RT_AM_CK and RT_AM_Inf. All experiments are characterized by three replicates except the RT_LCM series

RT_NFP_nsMyc_LCOs_6h	RT_NFP_sMyc_LCOs_6h	RT_NFP_s_nsMyc_LCOs_6h	RT_LCM_arbuscular	RT_LCM_cortical	RT_LCM_adjacent	RT_AM_CK	RT_AM_Inf
1.99 x 10^7	1.98 x 10^7	2 x 10^7	1.35 x 10^7	9.68 x 10^6	1.29 x 10^7	1.95 x 10^7	1.95 x 10_7
1.98 x 10^7	2 x 10^7	1.98 x 10^7	1.24 x 10^7	1.35 x 10^7	1.74 x 10^7	1.92 x 10^7	1.95 x 107
1.99 x 10^7	2 x 10^7	1.95 x 10^7	/	/	/	1.95 x 10^7	1.95 x 10^7

B) Sum of the expression values for experiments of [32]. Each experiment has three replicates

APP_P	NAP_C	ARB_A	CMR_K	EPI_E
1.52 x 10^5	1.46 x 10^5	1.18 x 10^5	1.13 x 10^5	1.15 x 10^5
1.50 x 10^5	1.55 x 10^5	1.16 x 10^5	1.11 x 10^5	1.15 x 10^5
1.37 x 10^5	1.46 x 10^5	1.22 x 10^5	1.11 x10^5	1.16 x 10^5

C) Sum of the expression values for experiments of [33]. Each experiment has three replicates

Meristem_root_nodD	Distal_infection_zone_root_nodD	Proximal_infection_zone_root_nodD	Infected_root_nodD	Uninfected_root_nodD
1.52×10^5	1.49×10^5	1.51×10^5	1.49×10^5	1.53×10^5
1.51×10^5	1.50×10^5	1.50×10^5	1.47×10^5	1.51×10^5
1.50×10^5	1.52×10^5	1.47×10^5	1.42×10^5	1.52×10^5
