# Supplementary material for: Cleaning the Medicago Microarray Database to Improve Gene Function Analysis
Source: Plants (Basel). 2021 Jun 18;10(6):1240. doi: 10.3390/plants10061240 (PMC8234645; doi:10.3390/plants10061240)
Supplement: Supplementary file 1 [file plants-10-01240-s001.zip › Table S06_Marzorati-Single-Hybridizations.pdf]

**Table S6: List of experiments in the MtGEA database with one single replicate**

| | | |
|---|---|---|
| Hyptl_A17_10C_35C_day1_1 | Hyptl_F83_10C_100C_day1_1 | Hyptl_A17_20C_100C_day1_1 |
| Hyptl_A17_10C_35C_day2_1 | Hyptl_F83_10C_100C_day2_1 | Hyptl_A17_20C_100C_day2_1 |
| Hyptl_F83_10C_35C_day1_1 | Hyptl_A17_20C_35C_day1_1 | Hyptl_F83_20C_100C_day1_1 |
| Hyptl_F83_10C_35C_day2_1 | Hyptl_A17_20C_35C_day2_1 | Hyptl_F83_20C_100C_day2_1 |
| Hyptl_A17_10C_50C_day1_1 | Hyptl_F83_20C_35C_day1_1 | RT_2wks_Sdl_Hydroponic_200mM_NaCl_0h_1 |
| Hyptl_A17_10C_50C_day2_1 | Hyptl_F83_20C_35C_day2_1 | RT_2wks_Sdl_Hydroponic_200mM_NaCl_1h_1 |
| Hyptl_F83_10C_50C_day1_1 | Hyptl_A17_20C_50C_day1_1 | RT_2wks_Sdl_Hydroponic_200mM_NaCl_2h_1 |
| Hyptl_F83_10C_50C_day2_1 | Hyptl_A17_20C_50C_day2_1 | RT_2wks_Sdl_Hydroponic_200mM_NaCl_5h_1 |
| Hyptl_A17_10C_100C_day1_1 | Hyptl_F83_20C_50C_day1_1 | RT_2wks_Sdl_Hydroponic_200mM_NaCl_10h_1 |
| Hyptl_A17_10C_100C_day2_1 | Hyptl_F83_20C_50C_day2_1 | RT_2wks_Sdl_Hydroponic_200mM_NaCl_24h_1 |
