# Supplementary material for: Cleaning the Medicago Microarray Database to Improve Gene Function Analysis
Source: Plants (Basel). 2021 Jun 18;10(6):1240. doi: 10.3390/plants10061240 (PMC8234645; doi:10.3390/plants10061240)
Supplement: Supplementary file 1 [file plants-10-01240-s001.zip › Table S07_Marzorati-Pearson-Under-Threshold.pdf]

**Experiment**	**Pair 1-2**	**Pair 2-3**	**Pair 1-3**
RT_Myc_3wks_infection	0.78	0.87	0.74
GiantCell	0.77	0.81	0.79
GallTissue_GiantCell	0.82	0.82	0.78
RT_LCM_arbuscular[1]	0.89	/	/
RT_LCM_cortical[1]	0.89	/	/
RT_LCM_adjacent[1]	0.87	/	/
Nod_Naut1_SalsC[2]	0.84	0.89	0.94
RT_CRR_72hpi[3]	0.99	0.91	0.89
RT_CRR_96hpi[3]	0.96	0.85	0.82
Root_A17_control[4]	0.99	0.84	0.85
HairyRoot_WT_Myc_CK[4]	0.99	0.84	0.85

Table S7: Groups of experiments showing Pearson correlation coefficients under a threshold value of 0.90. In red acceptable Pearson correlation coefficients. Values are approximated to the second decimal. We used a slash "/" to indicate that the experiment has only 2 replicates, in which case only one single pair of Pearson correlation coefficient could be calculated. Acceptable pairs are highlighted in red.
References: [1][31], [2][56], [3][57], [4][34]
